# Value of Including CT Chest in the Management of Acute Abdominal Emergencies: Experience During First Wave of COVID-19 Pandemic at a UK District General Hospital

**DOI:** 10.7759/cureus.19073

**Published:** 2021-10-27

**Authors:** Maitreyi S Patel, Jennifer S Jebamani, Shrabani Das Mohapatra

**Affiliations:** 1 General Surgery, Barking, Havering and Redbridge University Hospitals NHS Trust, Romford, GBR; 2 Surgery, The Princess Alexandra Hospital NHS Trust, Harlow, GBR; 3 General and Colorectal Surgery, The Princess Alexandra Hospital NHS Trust, Harlow, GBR

**Keywords:** lung base, acute abdominal emergencies, rt-pcr, covid-19, ct chest

## Abstract

Aims

COVID-19 can present with abdominal pain and affects the management of emergency surgical patients. The aim of this retrospective study was to assess the incidence of positive findings on CT chest in patients presenting with acute abdomen, who underwent CT thorax as part of the Intercollegiate General Surgical Guidance on COVID-19 during the first wave. To correlate CT chest findings with confirmed cases on reverse transcription polymerase chain reaction (RT-PCR), and to determine its influence on surgical management of abdominal emergencies.

Methods

A retrospective observational study of adult emergency surgical referrals (excluding trauma) for acute abdomen over a 10-week period was performed. COVID-19 changes on CT chest were categorized as per the British Society of Thoracic Imaging (BSTI) CT reporting criteria. Patient demographics, COVID-19 RT-PCR, management and outcome were recorded. Statistical analysis was performed using Microsoft Excel (Microsoft Corporation, Redmond, USA) with p-value significant at ≤0.05.

Results

Of the 160 patients included, 111 (69.38%) had COVID-19 RT-PCR. Twenty-four patients had CT chest findings suggestive of COVID-19. Amongst these, 45.83% demonstrated classic/probable CT features of COVID-19, of which 36.36% had positive RT-PCR. Most patients who had acute abdominal findings had a normal CT chest (p=0.03). Twenty-five (15.63%) patients presenting with abdominal pain had normal CT abdomen and seven (28%) of these had CT features of COVID-19. Only 43 (34.4%) patients needed a surgical intervention, of which 18.6% had COVID-19 changes on CT, confirmed by positive RT-PCR in 12.5%.

Conclusion

CT chest is an important investigation during the COVID-19 pandemic in suspected cases to help assess the severity of lung involvement. CT chest as an additional investigation modality in acute abdomen had clinically helped in triaging of patients to appropriate specialties but did not influence emergency surgical management.

## Introduction

December 2019 saw the emergence of a global pandemic caused by a highly infectious RNA virus the SARS-CoV-2. SARS-CoV-2 is highly homologous to SARS-CoV and shares the same cellular entry receptor, i.e angiotensin-converting enzyme 2 (ACE2) [1].

There have been reports of COVID-19 presenting with abdominal pain in the absence of respiratory symptoms [2]. A study by Pan et al reported that patients without gastrointestinal (GI) symptoms were more likely to recover and be discharged compared with those with GI symptoms (60% v/s 34%) [1].

The approximate rate of asymptomatic presentation of COVID-19 has been reported to be 17% [3]. A Chinese study by Lei et al demonstrated poor postoperative outcomes in COVID-19 positive patients undergoing surgery during the incubation period. This led to a rise in concerns regarding the higher postoperative intensive care unit admissions and mortality [4]. The SARS-CoV-2 infection and its sequelae consequently affect the management and outcomes of emergency surgical patients.

As the pandemic has progressed in the UK, several guidelines had been issued by multiple bodies, to include CT chest in surgical patients in order to assess for changes consistent with COVID-19 pneumonia. In March 2020, the British Society of Thoracic Imaging (BSTI) and the British Society of Gastrointestinal and Abdominal Radiology (BSGAR), produced a statement recommending CT examination of the chest in all patients undergoing CT for acute surgical abdomen [5]. This was later mirrored by the Intercollegiate General Surgical Guidance on COVID-19, which recommended any patient undergoing abdominal CT for acute abdomen should have a CT chest [6].

Approximately 4% of patients with COVID-19 infection present solely with GI symptoms, with others reporting some form of upper respiratory symptom [7]. With variations in sensitivity of thoracic CT in COVID-19 patients during the incubation period, the utility to include chest CT in all patients undergoing abdominal CT was questioned. This is affected by the prevalence of the disease, which varied greatly between different centers. We, therefore, conducted a retrospective analysis of non-traumatic emergency surgical referrals presenting predominantly with abdominal surgical complaints over a 10-week period from 25th March 2020 to 31st May 2020 at our centre, which is a District General Hospital.

The aim of this retrospective study was to assess the incidence of positive findings on CT chest in patients presenting with acute abdominal complaints, who underwent CT thorax as part of the guidelines in response to COVID-19 pandemic during the first wave, to correlate CT chest findings with RT-PCR, and determine the influence of including CT chest in the surgical management of acute abdominal emergencies.

This study was presented at the Association of Surgeons of Great Britain and Ireland (ASGBI) 2021 conference as a poster.

## Materials and methods

Study design

This was a single-centre retrospective observational cohort study.

Patients and data collection

All non-trauma emergency surgical patients aged ≥16 years with acute abdominal pain presenting to our hospital, which is a District General Hospital (DGH), who subsequently underwent CT chest, abdomen, and pelvis (CT CAP) as per the Intercollegiate General Surgical Guidance and BSGAR/BSTI statement, during the study period, 25th March, 2020 to 31st May, 2020, were included.

Patients were identified by searching for CT CAP scans undertaken during the study period using hospital's Computerized Radiology Information System (CRIS). CT CAP undertaken for trauma, elective investigation of surgical pathology, eg, staging of cancer, response to chemotherapy; and those for non-surgical pathology, were excluded from the analysis. Reports were reviewed for evidence of features of COVID-19 and graded into four categories: normal CT chest, classic/probable of COVID-19, indeterminate for COVID-19, and non-COVID-19 pathology based on the BSTI CT reporting criteria for COVID-19.

The CT findings were correlated with the reverse transcription-polymerase chain reaction (RT-PCR), presenting complaints, and blood results. RT-PCR results were obtained from the electronic patient records.

We further assessed the number of patients with normal CT abdomen in the presence of abnormal CT chest findings. Data on patient records and the clinical details was obtained from the hospital software Cambio COSMIC (Cambio Healthcare Systems A/S, Aarhus, Denmark).

The data on patient demographics, diagnosis, and management was obtained using COSMIC software. We recorded whether the patient was managed conservatively or required intervention, and the type of intervention required. Intervention was defined as surgery or non-operative intervention required for the management of surgical pathology.

Data interpretation and analysis

The data was collected on an Excel spreadsheet ( version 2007, Microsoft Corporation, Redmond, USA) stored on an NHS computer. No patient identifying data was included in the spreadsheet.

Continuous data were represented using the mean and standard deviation. Categorical variables were represented using percentages. Statistical analysis was performed using the t-test for continuous variables and Chi-square test for categorical variables. P-value≤0.05 was considered statistically significant. All statistical analyses were performed using Excel.

Ethical review

This retrospective audit received Institutional approval from the Clinical Audit department and informed consent was waived.

## Results

During the study, 262 adult patients underwent CT thorax abdomen and pelvis; 102 patients who underwent CT scans for trauma or alternative indications were excluded; 160 patients were included in the analysis.

Demographics

The mean age was 60.38 (SD 20.11) years; 45.63% (73) of the patients were females and 54.37% (87) males.

Presenting complaints

Abdominal pain was the most common symptom at presentation in 158 (98.75%) patients. Two patients presented with vomiting. Amongst the patients presenting with abdominal pain, 33 (20.88%) had associated vomiting and 5 (3.16%) had diarrhoea. The most common diagnosis was obstruction (19.38%) followed by appendicitis (11.88%) and diverticulitis (8.75%).

During the initial weeks of the pandemic, RT-PCR was done only in symptomatic patients, however, with the progression of the pandemic, RT-PCR was performed on all patients.

Total 47 (29.38%) patients had abnormal CT chest findings. The CT chest findings and RT-PCR results are summarized in Table 1. Due to the small number of patients with positive RT-PCR, the sensitivity and specificity of the test were not calculated.

**Table 1:** CT chest and RT-PCR results Positive CT chest findings and correlation with RT-PCR RT-PCR: reverse transcription polymerase chain reaction

CT chest scan	Total(n)	RT-PCR performed	RT-PCR positive
Classic/probable COVID-19	11(6.87%)	11(100%)	4(36.36%)
Indeterminate for COVID-19	13(8.13%)	11(84.62%)	0
Non-COVID-19	23(14.38%)	17(73.92%)	3(17.65%)
Normal	113(70.63%)	72(63.72%)	0
Total	160	111(69.38%)	7(6.31%)

Respiratory symptoms

Seven patients (4.3%) patients had respiratory symptoms on presentation. Three of the 11 patients with classic COVID-19 on CT had respiratory symptoms (27.27%), confirmed by positive RT-PCR in 66.67%. (Table 2).

**Table 2:** Respiratory symptoms and RT-PCR results

CT scan	Total	Respiratory symptoms present	Respiratory symptoms absent	Positive RT-PCR
Classic/probable COVID-19	11	3(27.27%)	8	2
Indeterminate for COVID-19	13	0	13	0
Non-COVID-19	23	2(8.69%)	21	0
Normal	113	2(1.77%)	111	0
Total	160	7(4.38%)	153	2

Lung base involvement in COVID-19-positive CT

A total of 8/11 (72.72%) patients with CT features of classic/probable COVID-19 had visible changes in the lung base, while 10/13 (76.92%) of patients who demonstrated indeterminate pattern on CT had lung base involvement.

Abdominal findings on CT CAP

Patients with positive abdominal CT were more likely to have normal CT chest (p=0.03) (Table 3).

**Table 3:** Abnormal abdominal CT *Chi-square test

CT scan	Positive abdominal scan	Negative abdominal scan	Total	
Classic/probable COVID-19	8	3	11	
Indeterminate for COVID-19	9	4	13	
Non-COVID-19	18	5	23	
Association with CT chest abnormality				
Total abnormal CT chest	35	12	47	p=0.03*
Normal	100	13	113	
Total	135	25	160	

Surgical management

One hundred and twenty-five of the 160 (78.13%) surgical referrals with abdominal pain had a surgical diagnosis. Of 125 patients managed by surgeons, 98 (78.4%) needed admission, while 27 (21.6%) were managed without admission. Amongst those admitted, five (5.10%) tested positive for COVID-19 and were admitted to the dedicated COVID-19 ward, where they were under joint care with the medical team.

Thirty patients were referred to the medical team for further management. Amongst these, four patients had positive abdominal findings on CT but were deemed unfit for any surgical intervention due to co-morbidities and frailty; hence, they were admitted under the medical team. These patients recovered with medical management and did not need surgical intervention. Three patients were referred to urology and two to gynaecology. The CT chest features of the patients managed primarily by the surgeons are summarized in Table 4.

**Table 4:** CT chest of surgical admissions

Surgical			Total=125
COVID-19 features on CT	Admission n=98	No admission=27	p =1
Classic/probable COVID-19	6 (6.12%)	2(7.41%)	
Indeterminate for COVID-19	9(9.18%)	0	
Non-COVID-19	10(10.20%)	5(18.52%)	
Normal	73(74.49%)	20(74.07%)	
Total	98	27	

Forty-three (34.4%) patients needed surgical intervention, of which 18.6% had COVID-19 changes on CT, confirmed by positive RT-PCR in only one (12.5%) case.

Eighty-two (65.6%) patients were managed conservatively. There was no significant difference in the presence of abnormal chest CT on surgical management of patients (Table 5). All patients with features of COVID-19 on CT chest were operated in separate theatres.

**Table 5:** Surgical management of patients *Chi-square test **Students t-test

Surgical n=126	Intervention N=43	Conservative N=82	Total N=125	p-value
Female	20	37	57	0.88*
Male	23	45	68	
Mean age	59.1	58.36		0.84**
Chest abnormality	43	82	125	
Classic/probable COVID-19	3	5		
Indeterminate for COVID-19	5	4		
Non-COVID-19	1	14		
Abnormal CT	9	23		0.38*
Normal	34	59		

**Figure 1:**
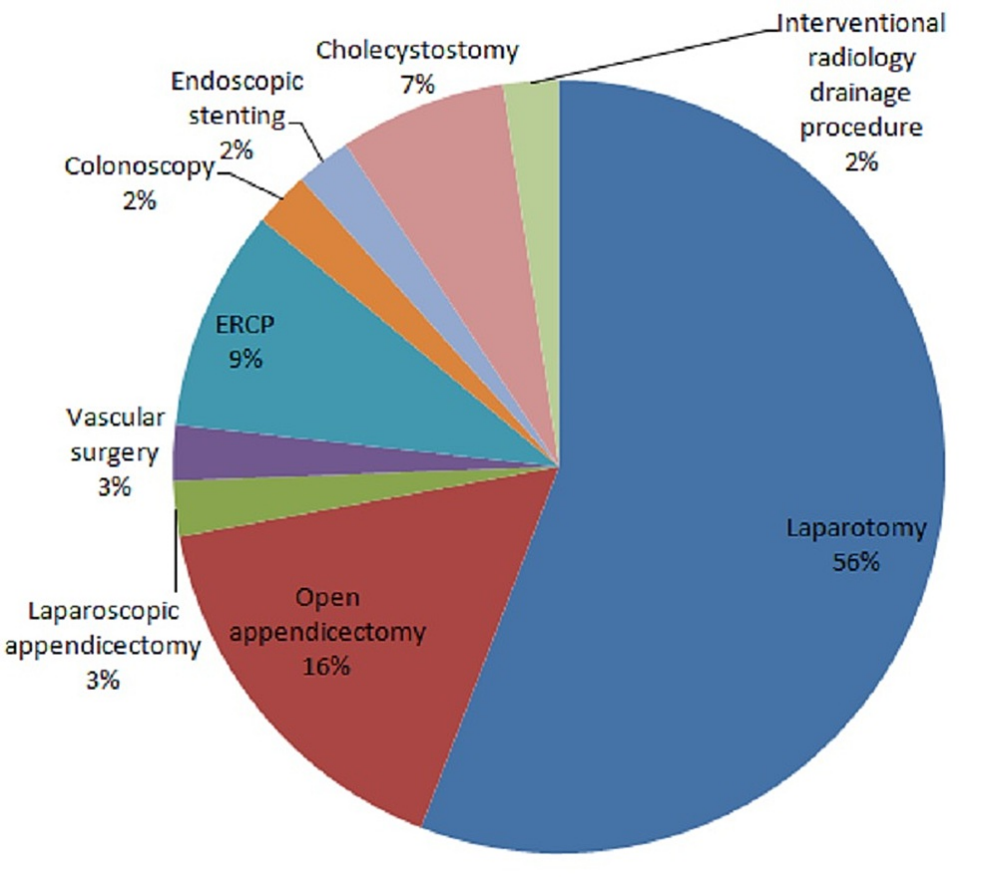
Pie chart depicting the surgical interventions

A total of 55.81% underwent a laparotomy while 16.28% had an open appendicectomy (Figure 1).

Twelve (7.5%) of the 160 patients had abnormal chest CT with a normal CT abdomen (Table 6). Of the 25 patients with normal CT abdomen, 12% had classic/probable COVID-19 pattern, 16% had indeterminate features of COVID-19 and 20% had a non-COVID-19 abnormality on CT chest. As depicted in Table 6, all patients had medical management.

**Table 6:** CT chest findings in patients with normal CT abdomen LFT: liver function test

Normal CT abdomen with chest CT findings	Classic/probable	Indeterminate	Non-COVID-19	Normal CT chest
n	3(12%)	4(16%)	5(20%)	13(52%)
Underlying lung pathology	1	0	1	2
Leucopenia	1	0	0	0
Lymphocyte count <1.0	2	2	3	1
Platelet <150	2	1	0	1
Deranged LFT	1	3	2	2
Surgical	Nil	2	2	8
Admission	2	4	4	7
Management	Medical	Medical	Medical	Medical

## Discussion

COVID-19 is most commonly associated with respiratory symptoms, however, as the pandemic has progressed, additional symptoms related to the disease have been identified, including headache, flank pain, abdominal pain, diarrhea, and vomiting [8]. Approximately 4% of patients with COVID-19 infection present solely with gastrointestinal symptoms [7]. Abdominal and back pain has long been described as a symptom in pneumonia secondary to pleural irritation [9]. In some patients, abdominal pain is the dominant symptom without obvious respiratory symptomatology [10]. However, abdominal pain alone is relatively rarely reported, while anorexia and diarrhea are the most commonly reported gastrointestinal symptoms, followed by nausea and vomiting [11]. Most patients, i.e., 158, in our study presented with abdominal pain, and only eight of these patients had respiratory symptoms along with abdominal pain at presentation. Diarrhoea and vomiting were present in 3.16% and 20.8% of patients respectively. Of the seven COVID-19-positive patients, three patients (42.8%) presented with vomiting and abdominal pain. This difference could be attributed to the fact that the number of COVID-19-positive patients in our study was low hence it is not possible to estimate the correct incidence of gastrointestinal symptoms in our study cohort. There was no distinct pattern of abdominal symptoms observed in our study that seemed specific for COVID-19.

Due to the variability of symptoms and atypical symptoms at presentation, it is important to obtain a detailed history and detailed examination to be able to further stratify the possibility of COVID-19 as the cause of symptoms. The presence of respiratory symptoms or history of close contact with an individual with SARS-CoV-2 should prompt further investigations which includes the addition of CT chest to CT abdomen.

The reported sensitivity of CT is in the range of 93% to 98%, with specificity being between 25% to 53% [12,13]. In these studies, the patients were symptomatic or had positive RT-PCR, which is different from our patient cohort and could explain the poor correlation of CT features of COVID-19 with RT-PCR in our study, as would the limitation imposed by the small sample size. Although specific, RT-PCR testing has a reported sensitivity of only 60-70% [14]. Additionally, the method of performing the test may result in variation in the result.

With a high reported prevalence of lower-lobe abnormalities identified in CT in patients with COVID-19 in both Asian and European populations [15], there have been reports that assessment of lung bases in CT abdomen alone could suffice. In our study, we found that 27.27% (three out of 11) patients with classic/probable COVID-19 CT chest findings had a sparing of the lung bases which may possibly result in missing COVID-19. However, the low number of COVID-19 positive patients in our study makes it difficult to make a recommendation regarding the use of lung base assessment alone versus a whole chest CT for assessment of COVID-19.

The high number of surgical diagnoses in our cohort of patients (125/160, i.e., 78.13%) corroborates the understanding that abdominal pain is a relatively rare reported symptom associated with COVID-19. Hence, patients who were emergency surgical referrals would be more likely to have had an abdominal pathology.

Bhayana et al. [16], reported abdominal CT findings in COVID-19 patients and found bowel wall abnormalities (primarily bowel wall thickening) in 31% and pneumatosis intestinalis or portal venous gas in almost 10% of the 42 abdominal CT scans performed. We did not find any of these abnormalities in our study cohort. A possible explanation for this difference could be that they had included inpatients that had tested positive for COVID-19 in their study and reviewed their abdominal imaging, while our study evaluated the CT findings of the patients at presentation including those who did not have COVID-19 positive results.

There was no significant difference in surgical admissions due to abnormal CT chest features. This data demonstrates that emergency surgical admissions would not be influenced by additional findings on CT chest.

Our results demonstrate no significant correlation of abnormal CT chest findings with emergency surgical intervention. A similar report has been published by Chetan et al. [17], where they found no change in the surgical management in the acute abdominal cohort. In our study, a higher number of patients, i.e., 33 out of 43 (76.74%), underwent surgery as compared to non-operative intervention, i.e. nine out of 43 (20.9%).

Further, 20.93%, i.e., nine out of 43, had no RT-PCR done prior to intervention; however, seven of these nine patients underwent emergency surgery, demonstrating the fact that emergency surgery was not affected by RT-PCR results. Additionally, all procedures were performed using full personal protective equipment (PPE) irrespective of COVID-19 results.

Leucopenia and lymphopenia is a well-described laboratory finding in COVID-19. It is possible that the impaired immune system due to COVID-19 makes these patients more susceptible to other infectious processes [8]. In addition to digestive symptoms, patients with COVID-19 are also at risk of developing liver injury. Studies have shown that patients had varying degrees of liver function abnormalities - the incidence ranged from 15% to 53% mainly indicated by abnormal transaminase concentrations, accompanied by slightly increased bilirubin concentrations [18]. The small cohort size limits the assessment of the utility of these blood tests for the assessment of COVID-19 in our study.

In May 2020, BSGAR-BSTI updated their statement and advised that the need for CT chest has diminished due to decreasing prevalence of COVID-19 and improved availability of RT-PCR [5].

With the widespread availability of RT-PCR, whole CT chest should be sparingly used in the assessment of acute surgical patients. Although low, 7.5% of patients in our study did have positive CT chest findings in absence of acute abdominal CT findings. However, negative CT abdomen should prompt further investigations to rule out COVID-19 in acute surgical referrals, to help diagnose occult COVID-19. This can help manage the patients in separate wards and ensure staff safety. Our results do illustrate that the clinical presentation of COVID-19 encompasses a diverse spectrum of presentations including atypical presentations like abdominal pain, and clinicians and radiologists should be aware of these. With the increasing prevalence of the disease and the rising number of asymptomatic carriers, surgeons are more likely to encounter these patients presenting with abdominal complaints who may have incidental pulmonary findings of COVID-19 on CT.

We acknowledge that our study is not without limitations such as a small sample size.

This study is further limited by retrospective analysis of data from only a single centre, which reflects the prevalence of SARS-CoV-2 in our study population. This potentially means that our study findings may differ from other centers with different COVID-19 disease burden relating to their prevalence rate. The centers with a higher prevalence of COVID-19 infection may experience a greater diagnostic yield than that seen in our cohort. Not all patients had RT-PCR tests during the initial few weeks of the study period as only symptomatic patients were being tested. Furthermore, there may be inter and intra-observer variations in CT reporting of COVID-19 findings.

However, despite these limitations, this study does give a practical overview of general surgical emergency management, and we believe the results should be reproducible.

## Conclusions

Inclusion of CT chest in acute abdomen helped to identify occult COVID-19. Early identification of COVID-19 can help to prevent disease transmission by prompt isolation and use of personal protective equipment in the management these patients. CT chest as an additional investigation modality in acute abdomen had clinically helped in triaging of patients to appropriate specialties but did not influence emergency surgical management.
